# YY1 Is a Key Player in Melanoma Immunotherapy/Targeted Treatment Resistance

**DOI:** 10.3389/fonc.2022.856963

**Published:** 2022-06-01

**Authors:** Dominika Kwiatkowska, Ewelina Mazur, Adam Reich

**Affiliations:** Department of Dermatology, Institute of Medical Sciences, Medical College of Rzeszow University, Rzeszow, Poland

**Keywords:** malignant melanoma, immunotherapy, treatment resistance, YY1, PD1

## Abstract

Malignant melanoma, with its increasing incidence and high potential to form metastases, is one of the most aggressive types of skin malignancies responsible for a significant number of deaths worldwide. However, melanoma also demonstrates a high potential for induction of a specific adaptive anti-tumor immune response being one of the most immunogenic malignancies. Yin Yang 1 (YY1) transcription factor is essential to numerous cellular processes and the regulation of transcriptional and posttranslational modifications of various genes. It regulates programmed cell death 1 (PD1) and lymphocyte-activation gene 3 (LAG3) by binding to its promoters, as well as suppresses both Fas and TRAIL by negatively regulating DR5 transcription and expression and interaction with the silencer region of the Fas promoter, rendering cells resistant to apoptosis. Moreover, YY1 is considered a master regulator in various stages of embryogenesis, especially in neural crest stem cells (NCSCs) survival and proliferation as it acts as transcriptional repressor on cancer stem cells-related transcription factors. In addition, YY1 increases the metastatic potential of melanoma through negative regulation of microRNA-9 (miR-9) expression, acts as a cofactor of transcription factor EB (TFEB) and contributes to autophagy regulation, mainly due to increased transcription of genes related to autophagy and lysosome biogenesis. Therefore, focusing on the detailed biology and administration of therapies that directly target YY1 or crosstalk pathways in malignant melanoma could facilitate the development of new and more effective treatment strategies and improve patients’ outcomes.

## Introduction

Malignant melanoma is one of the most aggressive types of skin malignancy, presenting high potential to form metastases. The incidence of melanoma has significantly increased all over the world within the last decades (1). Many factors can contribute to the malignant transformation of melanocytes and melanocytic lesions, among which one can distinguish a complex interaction between environmental and genetic factors (2). Oncogenic mutations in genes are widely observed in melanoma, with MEK, BRAF, NRAS, and NF1 being the most common ones (2).

The first step of the therapeutic management of cutaneous melanoma is surgical excision of the primary lesion with adequate margins. In a case of melanoma *in situ* or very thin melanomas (<0.8 mm), surgery is considered sufficient, whereas patients with more advanced stages often require adjuvant therapy (3). Malignant melanoma is a neoplasm which often shows resistance to standard cytotoxic treatments. However, it demonstrates a high potential for induction of a specific adaptive anti-tumor immune response being one of the most immunogenic malignancies (4). In recent years, the knowledge regarding the molecular biology of malignant melanoma has been expanding, and, thus, a huge progress in the field of immunotherapy (with its many innovative therapeutics) in the treatment of malignant melanoma has been observed. However, achieving disease remission or at least stabilization in patients with poor response to immunotherapy is almost impossible. Therefore, a better understanding of processes by which malignant cells evade immune surveillance is needed to increase treatment efficacy or reverse resistance. Recently, the possible role of the Yin Yang 1 (YY1) transcription factor in the pathogenesis and drug resistance of malignant melanoma is considered.

YY1, also known as a nuclear factor-E1 (NF-E1), is a GLI-Kruppel class of zinc finger protein transcription factors (5). YY1 is ubiquitously expressed, and its expression is on the same level within all tissues of the human body (6). This multifunction protein is essential to numerous cellular processes and the regulation of transcriptional and posttranslational modifications of various genes (7–9). The transcription factor YY1 has been studied in many cancers. To date, it was shown that YY1 exerts a role in tumorigenesis, mainly through alteration in cell cycle signaling pathways, the process of epithelial-mesenchymal transition, resistance to immunotherapy and targeted therapy, as well as the formation of metastasis (10–13). Moreover, the role of YY1 in modulating chronic inflammation, apoptosis evasion, angiogenesis, and genome instability have been previously described in the literature (14–16).

In the present review, we have highlighted the importance of the overexpression of the transcription factor YY1 in malignant melanoma tumorigenesis and its possible role in tumor surveillance and treatment resistance.

## The Structure and Function of Yin Yang 1 Protein

The human YY1 consists of 414 amino acids and was first described as a transcriptional repressor that interacts with the P5 promoter for the adeno-associated virus (5, 17). However, YY1 can either positively or negatively regulate its target genes, acting as both a transcriptional activator and repressor. YY1 recognizes and binds to specific DNA sequences through the four zincs fingers, forming the DNA-binding domain (18). Both DNA binding-dependent and independent functions of YY1 have been already described. A recent study showed that YY1 also controls gene expression by binding to active enhancers and promoter-proximal elements (19). Furthermore, the pattern of this control varies due to the many interactions with versatile proteins. YY1 can also act indirectly, by affecting the chromatin state *via* the recruitment of histone methylases, acetylases, and deacetylases (20–22). These findings could enlighten the variety of YY1’s functions including gene activation and repression. YY1 might impact positive or negative effects on transcription by influencing the positioning of other transcription factors in enhancers and promoters.

## YY1 and Immune Cells

The expression of YY1 influences the development of B and T cells on every stage (23). Germinal centers are vitally important for humoral immunity. During germinal center development, B cells depend strictly on YY1 expression (24). It was shown that the deletion of the YY1 gene reduces the amount of germinal center B cells and decreases survival. Interestingly, YY1 possesses dual functions, either as an activator or repressor. YY1 induces Th2 cells differentiation and, contrarily, blocks Treg cell differentiation and function. To control Th2 cytokine genes, YY1 mediates chromatin remodeling and chromosomal lopping of the Th2 cytokine locus (25). The primary function of Treg cells is the prevention of excessive immune response through inhibition of differentiation and proliferation of other T cells subpopulations, such as Th1, Th2, Th17, and Tfh cells (26). YY1 inhibits the differentiation and function of Treg cells by blocking Foxp3 – a transcription factor for Treg cells (27). Since melanomas are infiltrated by large numbers of Tregs to evade immune response, downregulation of the Treg cells’ function in tumor microenvironment may enhance protective immunity against this cancer. On the other hand, the YY1 seems to promote T cell exhaustion by repressing IL-2 and upregulating checkpoint inhibitors. Exploring the exact role of YY1 on the molecular riddles of the melanoma microenvironment may shed light on new and better treatment strategies against this cancer.

## Malignant Melanoma Evades and Inhibits Immune Responses

Malignant melanoma may evade the host’s cellular regulatory and immune system. Chronic inflammation can lead to exhaustion of the immune system, and inhibitory receptors and their ligands play a crucial role in this process (28). The persistent antigen exposure by melanoma cells is dependent on the up-regulation of immune checkpoints, such as programmed cell death 1 (PD1), cytotoxic T lymphocyte antigen 4 (CTLA4), T-cell immunoglobulin, and mucin-domain containing-3 (TIM3) or lymphocyte-activation gene 3 (LAG3) and results in negative feedback for the cytotoxic T-cells. Thus, blocking the different immune checkpoints has become a cornerstone of modern immunotherapy and the usefulness of CTLA-4, PD-1 and PD-L1/2 blockers in advanced melanoma therapy has been most widely studied to date (29, 30). However, there is still a large group of patients in whom treatment with checkpoint inhibitors proved to be unsuccessful. In addition, prolonged treatment with the same medication could potentially lead to cross-resistance to a variety of therapies.

## Regulation of PD1 and LAG3 by YY1

**Figure 1:**
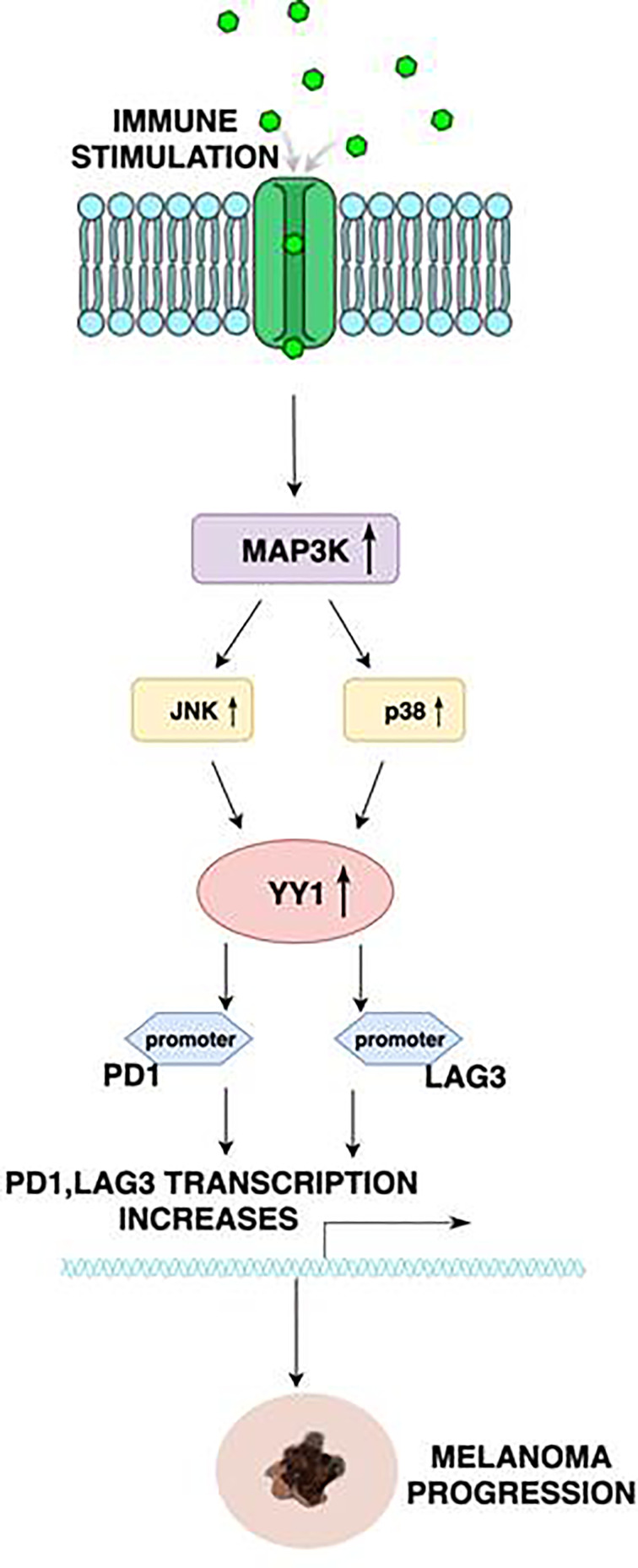
Schematic diagram illustrating the regulation of PD1 and LAG3 by YY1. Stimulation of receptors on the immune cells results in the activation of the MAP3K, leading to the upregulation of JNK and p38. p38 and JNK, in turn, activate YY1. YY1 bind to the PD1 and LAG3 promoters to initiate transcription. This mechanism is correlated with the exhaustion of tumor infiltrating T lymphocytes, and thus assists melanoma progression.

Interestingly, YY1 regulates PD1 and LAG3 by binding to the promoters of PD1 and LAG3. In the study provided by Balkhi et al. (31), mutation of the YY1’s binding sites elicits increased transcription with repeat T-cell stimulation, which proves that both PD1 and LAG3 are positively regulated by YY1 (**Figure 1**). In melanoma, functional exhaustion with increased fractions of PD1+ CD4 cells were found to be associated with a high level of YY1. On the other hand, downregulation of YY1 in repeatedly stimulated cells causes a reduction of PD1 and LAG3. Moreover, in the aforementioned study, 15 human melanoma samples and 10 normal skin biopsies were examined. Exhaustion of tumor-infiltrating lymphocytes was mostly tied with overexpression of PD1, along with YY1-cofactor Ezh2. In normal skin, no exhaustion markers were found. Human melanoma sections stained by immunofluorescence showed activation of the p38MAPK/JNK pathway in tumor-infiltrating lymphocytes, which drives YY1 expression, leading to PD1 upregulation. These findings significantly improved our knowledge of the relevance of T-cell activation through a p38MAPK/JNK/YY1 pathway.

The specific tumor microenvironment uses several mechanisms to defend itself from cell death mediated by cytotoxic T-cells (32). One of them is an upregulation of PD-L1 expression. Multiple pathways have been identified by which YY1 could be involved in regulating PD-L1 expression. Recent findings indicate that therapies directly targeting YY1 or cross talk pathways could improve patients’ outcomes due to the downregulation of PD-L1 expression on tumor cells.

## YY1 Regulates Drug Resistance in Melanoma

**Figure 2:**
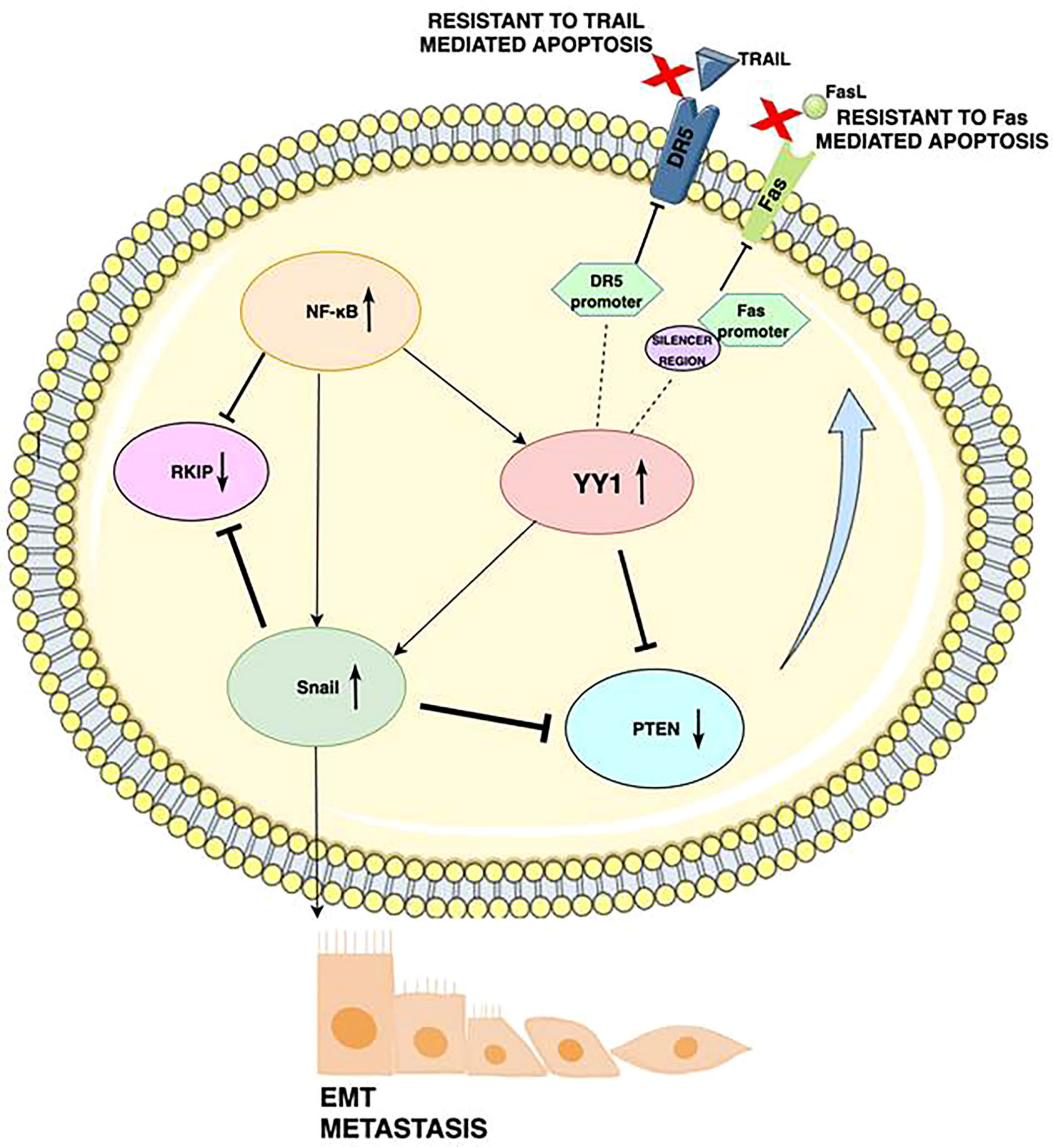
Schematic diagram illustrating the mechanism by which YY1 regulates drug resistance in cancers: Activation of the YY1 negatively regulates DR5 transcription by binding to the DR5 promoter region, which elicits resistance to TRAIL-induced apoptosis. YY1 binds to the silencer region of the Fas promoter, blocks Fas expression and further leads to downregulation of Fas expression and resistance of cancerous cells to Fas-mediated apoptosis. High NF-kB expression corresponds to low RKIP expression, as well as NF-kB induces YY1 expression, which then represses PTEN. The underexpression of Fas is also caused by NF-κB’s activation of YY1. YY1 induces SNAIL, the repressor of RKIP. Expression of YY1 and Snail is participating in downregulation of PTEN and DR5. As a result, YY1 promotes epithelial to mesenchymal transition (EMT) and metastasis through upregulation of Snail.

During tumorigenesis, pathways that regulate cell survival and apoptosis, become dysregulated. Bonavida et al. proposed a model of a dysregulated circuit consisting of the NF-κB/Snail/YY1/PTEN/RKIP loop, which may be crucial for melanoma cell growth, survival, and drug resistance (33, 34). Upon activation, the NF-κB signaling pathway (via autophagosomes, p62 and JNK signaling leading to the expression of their target genes HIF-1α, IL-8, BCL2 and BCLXL) regulates the transcription of YY1 and Snail 1 (35). In response to stimulation, these target genes further negatively regulate the phosphatase, and tensin homolog (PTEN), and RKIP which in turn activates the AKT/mTOR-C1 pathway and its interaction with LC3. Contrarily, the induction of RKIP downregulates NF-κB signaling and thus inhibits YY1 and Snail 1. At the same time, the expression of PTEN inhibits the PI3K/AKT (phosphoinositide-3-kinase/AKT) pathway (**Figure 2**) leading to a significant inhibition of AKT-mediated signaling pathway, but also to autophagy proteins such as LC3-I/II, LAMP, cathepsin B, and PI3K-AKT-MTOR. Increased PI3K/AKT activity appears to have a major role in resistance to BRAF inhibitors (36).

The regulation of various processes in cancer cells is strongly dependent on the different expression levels of each of the gene products. There is evidence revealing that YY1 is involved in melanoma resistance to apoptosis (37, 38). The extrinsic pathway of apoptosis involves death ligands on the surface of cytotoxic cells, which bind corresponding receptors on target cells. The molecular pathways by which Fas and TRAIL activate both the extrinsic and intrinsic apoptosis pathways have been well characterized (39). However, the exact mechanisms of resistance to TRAIL and Fas have not yet been fully elucidated. YY1 may play a role in this process, as YY1 can suppress both Fas and TRAIL, rendering cells resistant to apoptosis. Expression of Fas is negatively regulated by the interaction of YY1 with the silencer region of the Fas promoter (40). In another mechanism, YY1 negatively regulates DR5 transcription and expression, inducing resistance to TRAIL-induced apoptosis (41). Fas and TRAIL-mediated apoptosis can be restored by inhibiting YY1, which can reverse the resistance to many drugs.

The studies of new and more effective treatment strategies for melanoma are of great interest as still many patients with advanced melanomas demonstrate poor outcomes. As already mentioned, YY1 strongly contributes to the pathogenesis of this malignancy. The research above describes numerous melanoma oncogenes, several of which seem to be a good target for future therapies. Patients with melanoma may benefit from the inhibition of EMT. This approach aims to target miRNAs to downregulate YY1 expression and result in the inhibition of EMT in cancer cells (42). Previous findings demonstrated that YY1 is inhibited by nitric oxide. In the NF-κB/SNAIL/YY1/RKIP/PTEN loop, nitric oxide donors downregulate YY1 through S-nitrosylation and subsequently lead to inhibition of EMT. Inhibiting YY1 by nitric oxide may also sensitize melanoma cells to immunotherapies, like Fas-L, TRAIL, and anti-PDL1-dependent treatment (32, 41, 43). Moreover, YY1 activity is reduced significantly by rituximab, which results in up-regulation of Fas-induced apoptosis. Therefore, combined treatment with YY1 inhibitors may help overcome the problem of tumor cells’ resistance (39). Finally, YY1 promotes melanoma angiogenesis, acting as a positive regulator of VEGF. A therapeutic intervention involving this mechanism may reduce neoangiogenesis and tumor growth

Still, many questions arise regarding the exact role of YY1 in melanoma pathogenesis, progression, and drug resistance. A deeper characterization of YY1 molecular pathways of action may help define novel biomarkers useful in the prognosis of melanoma.

## YY1 and Cancer Stem Cells

Malignant melanoma originates from differentiated melanocytes that arise from neural crest stem cells (NCSCs) (44). However, a multitude of biochemical data suggests that it could also originate from cancer stem cells (CSCs) called tumor-initiating cells (TICs). CSCs are pluripotent cells with self-renewal potential, allowing cell proliferation without losing its differentiation potential. They are proved to be associated with worse clinical outcomes. The multilineage differentiation abilities can contribute to melanoma diversity and heterogeneity (45, 46). Assuming that melanoma formation is related to the CSC population, identifying molecular targets in these cells could help to provide new treatment modalities. It seems that YY1 may be associated with melanoma CSCs.

Overexpression of many transcription factors such as SRY-Box Transcription Factor 2 (SOX2), octamer-binding transcription factor 4 (OCT4), B lymphoma Mo-MLV insertion region 1 homolog (BMI1), and Nanog Homeobox protein (NANOG) have been identified as key players in CSCs-related tumor initiation and metastasis formation. Interestingly, YY1 expression may also depend on Sox2, OCT4, BMI1, and NANOG’s activities (47). The study by Kaufhold et al. (47) has identified YY1 as a possible transcriptional repressor that acts on CSCs-related transcription factors. The correlation between expression levels of YY1 and Sox2 was observed among all cancer types. Subsequently, the four groups of cancers were distinguished. The highest expression of YY1 and Sox2 proteins represented the group assembled by melanoma, colorectal cancer, and lymphomas. In addition, the study showed the presence of putative YY1 binding sites on all regulatory regions of the transcription factors. However, none of the putative transcription binding sites for BMI1, SOX2, and OCT4 were found on the YY1 or each other’s regulatory regions, suggesting a multi-dynamic regulatory control of expression.

The invasiveness of malignant melanoma is attributed by some authors to SOX2 overexpression. Conversely, recent studies revealed that malignant melanoma initiation and progression are not affected by complete inhibition of SOX2 function (48). It can be explained by different study designs, as well as the aforementioned melanoma heterogeneity. Another set of data suggests that melanoma formation *in vitro* and *in vivo* is counteracted by the inactivation of neural crest stem cell factor SOX10. Furthermore, a subset of genes bound by YY1 is also co-bound by SOX10, which strongly suggests a connection between YY1-dependent metabolic processes and SOX10 (48). YY1 is considered a master regulator in various stages of embryogenesis, especially in NCSCs survival and proliferation.

YY1 plays a crucial role in metabolic reprogramming that converts a normal melanocyte into a melanoma-competent cell. This transition is achieved *via* binding and regulation of a subset of genes responsible for glucose metabolism, mitochondrial electron transfer chain, tricarboxylic acid cycle, one carbon metabolism, nucleotide metabolism, and protein synthesis. Varum et al. have found that the loss of YY1 leads to, among others, a reduction in basal oxygen consumption rates, maximal respiratory capacity, and ATP turnover, essential for cancer proliferation. These findings were especially expressed in melanoma cells, suggesting their particular sensitiveness to YY1 knockdown. They also reported that YY1 not only co-binds in different combinations with SOX10 and MITF (Melanocyte Inducing Transcription Factor) but also controls a MITF and c-MYC-regulated gene set. Therefore, it affects cancer stem cells metabolism in melanoma (49, 50).

## The Role of YY1 in Tumor Angiogenesis

**Figure 3:**
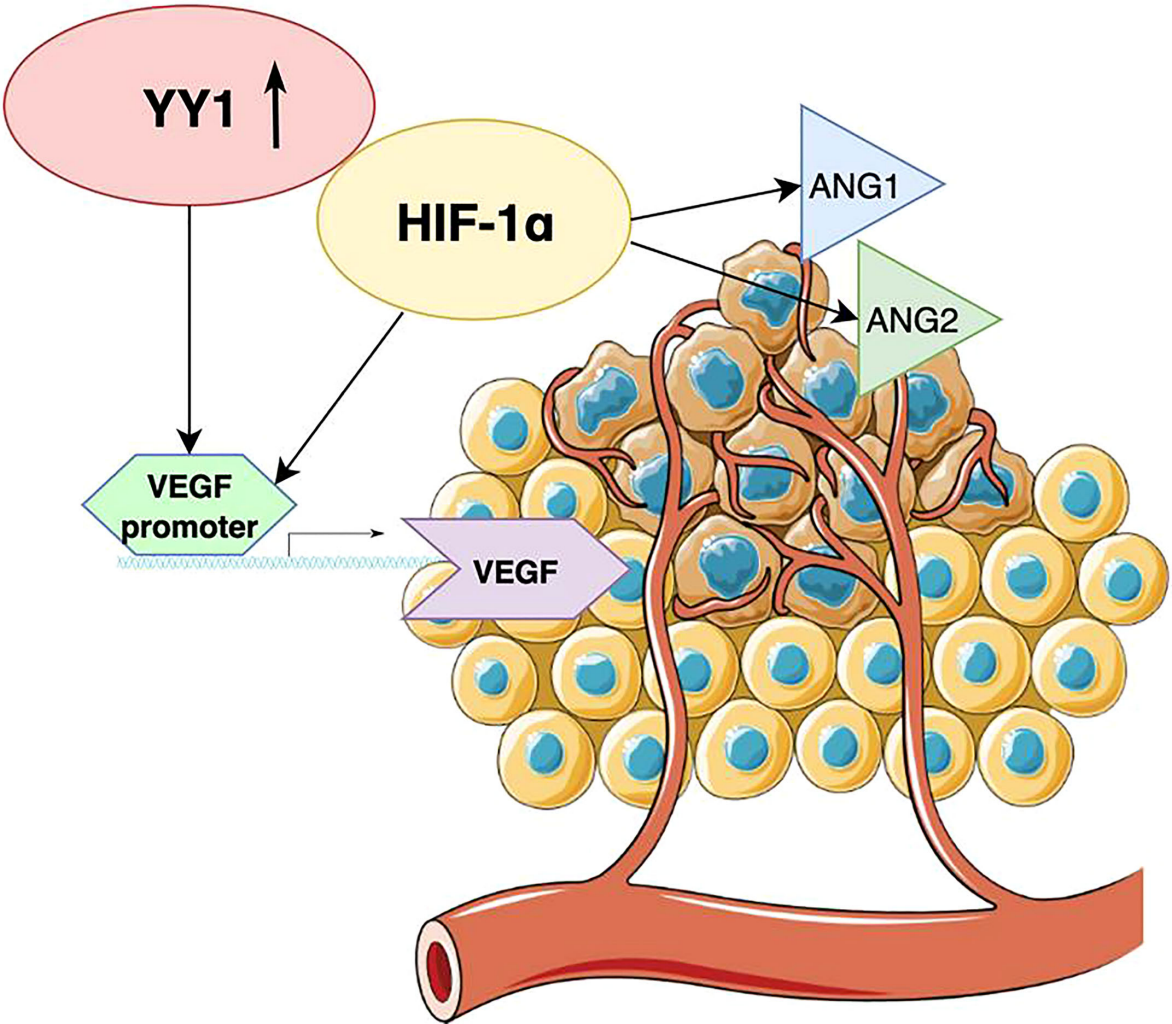
Schematic diagram illustrating the role of YY1 in tumor angiogenesis. YY1 regulates the increase of VEGF by suppressing HIF-1α, which also upregulates the expression of angiopoietin factors ANG-1 and ANG-2.

Angiogenesis is crucial in the growth of various cancers, including malignant melanoma. This process allows to supply nutrients and oxygen to tumor cells. Studies have shown that without angiogenesis, the growth of a tumor larger than 1 mm is restricted, as diffusion of oxygen from blood vessels is not sufficient (51). To date, the family of vascular endothelial growth factors (VEGFs) turned out to be the major mediator of tumor angiogenesis. VEGF family consists of structurally related molecules including VEGF-A, VEGF-B, VEGF-C, VEGF-D, among which VEGF-A is typically referred to as VEGF (52). Through interaction with the VEGF receptor (VEGFR), VEGF promotes endothelial cell proliferation, which further mediates sprouting of angiogenesis. YY1 may participate in this process through positive regulation of VEGF transcription (53, 54). Moreover, YY1 regulates the increase of VEGF by suppressing HIF-1α proteasomal degradation which causes its accumulation (**Figure 3**). HIF-1α plays a pivotal role in the adaptation of cancer cells to hypoxia, as well as promotes tumor angiogenesis through contribution to endothelial cell proliferation and migration (55). A fraction of cells that can survive hypoxia exhibit a more invasive phenotype and refraction to anticancer therapies. In another mechanism, YY1 can influence tumor angiogenesis by increasing the expression of angiopoietin factors ANG-1 and ANG-2, which are mediators of microvascular remodeling and capillary maturation (**Figure 3**). Current findings suggest that YY1 regulates the expression of angiogenic genes that are critical for proper vascular development and maintaining homeostasis. The study conducted by Zhang et al. showed that endothelial cell specific YY1 deletion in mice causes embryonic lethality due to abnormal angiogenesis (56). Extensive research also revealed that YY1 deletion in the tamoxifen-inducible endothelial cell-specific YY1 deficient mouse model inhibited angiogenesis and the melanoma growth (57). A high level of YY1 expression was noted in tumor cells and tumor-associated endothelial cells in human melanoma tissues. These findings suggest that silencing endothelial YY1 could decline melanoma angiogenesis and thus can be a potential effective treatment option. Future research should focus on the usefulness of nitric oxide and rituximab to decrease tumor angiogenesis, as those drugs can potentially inhibit YY1 expression (39, 40).

## YY1 and Melanoma Metastasis

Tumor metastasis is an intricate process involving various signaling pathways. Genetic alterations enable the cancer cells to acquire the biological properties essential for forming distinct metastasis. Zhao et al. discovered that YY1 increases the metastatic potential of melanoma through negative regulation of microRNA-9 (miR-9) expression (58). MiR-9 acts as a NF-κB-Snail1 pathway suppressor, therefore, inhibits migration and invasion of malignant melanoma cells (59, 60). MiR-9 directly binds to RING1 and YY1 binding protein (RYBP) 3′ untranslated region (3’UTR), thus leading to mRNA degradation. These findings demonstrated a potential role of RYBP as an oncogene by identification of a novel and vital YY1 ~ miR-9 ~ RYBP axis (58).

YY1 expression is closely related to tumor metastasis. YY1 directly binds the snail 3’ enhancer, which regulates snail transcription. To change the chromatin structure of the 3’ enhancer, YY1 participates in the recruitment of histone modification enzymes. It is well known that the migratory capacity of cancers is defined by snail overexpression. The regulation of the Snail gene is crucial for the epithelial-mesenchymal transitions (EMT), a process in which epithelial cells acquire invasive properties by transformation into migrating mesenchymal cells. EMT is essential for cancer invasiveness. As mentioned before, YY1 is an integral part of the NF-κB/Snail/YY1/RKIP axis, which in turn is the positive EMT regulator. However, the exact regulation of the *Snail* gene by the YY1 is not yet fully understood.

## YY1 and Autophagy

The importance of autophagy in the regulation of cancer cell biology and its response to various therapies is now intensively investigated. Autophagy is an intracellular degradative process. This mechanism requires the formation of autophagosomes and then fusion with lysosomes. Some studies to date have shown that the resistance of cancer cells to anticancer drugs can increase through the upregulation of autophagy (61, 62). It was recently introduced that YY1 acts as a cofactor of TFEB and contributes to autophagy regulation, mainly due to increased transcription of genes related to autophagy and lysosome biogenesis (63, 64). These authors also investigated whether YY1 mediated autophagy could modulate the activity of one of the commonly used in melanoma patients BRAF inhibitors – vemurafenib (63). Interestingly, the research showed that suppression of YY1 contributes to increased antitumor efficacy of vemurafenib (63).

Therefore, YY1 directs the apoptosis-anti-apoptosis balance in favor of tumor cells survival, protecting the cells from damage and activating autophagy-mediated survival (65). Protein kinase inhibitor - sorafenib was shown to block human receptor tyrosine kinase (c-Kit), platelet-derived growth factor receptor (PDGFR), vascular endothelial growth factor receptor (VEGFR), BRAF, and c-RAF signaling pathways both *in vitro* and *in vivo*. It prevents the tumor growth of hepatocellular, breast, and pancreatic cancer types and suppresses tumor growth and metastasis in melanoma by c-Kit inhibition (66). Interestingly, sorafenib may sensitize melanoma to vemurafenib through activation of oxidative stress (upregulated reactive oxygen species production, malondialdehyde, and iron, but decreased glutathione concentration). Moreover, sorafenib strongly promoted vemurafenib-mediated cell death (67).

## YY1 as a Prognostic Marker of Melanoma

YY1 is characterized by a multifunctional mechanism of action, as it can act as an initiator, activator, or repressor of transcription. Increased levels of YY1 have been observed in both primary and metastatic melanoma (58). The overexpression of YY1 might be associated with melanoma progression, as it is involved in many biological processes, including modulation of the immune response, apoptosis, epithelial to mesenchymal transition, angiogenesis, and metastasis formation. Given that YY1 is essential for many biological processes, it may be an interesting prognostic target for melanoma.

Bioinformatics analyses on the expression of YY1 in melanomas seem to confirm the relevant role of this transcription factor in melanoma development and progression (68). It was revealed that an increased level of YY1 positively correlates with mRNA expression of the nectin like molecule-5 (*NECL-5*) gene (69). NECL-5 is a cell adhesion molecule, the decrease of which reduces migration, proliferation, and metastasis of cancer cells. The study conducted by Bevelacqua et al. suggests that NECL-5 may be a potential marker of melanoma progression (69). Additional bioinformatics studies also suggested an association between YY1 and MITF in melanoma progression (70). MITF activity strongly influences the phenotype of melanoma cells and determines its invasiveness (70). YY1 is one of the transcription factors that regulate MITF-dependent genes. Moreover, both MITF and YY1 are localized in the area of promoters of genes important in melanocyte differentiation. Besides MITF, transcription factor activator protein 2 A (TFAP2A) and SOX10 have also been identified to be localized alongside YY1. Furthermore, MITF interacts with PBAF (polybromo-associated BRG1/BRM-associated factor) chromatin remodelling complex comprising BRG1 and CHD7 (chromodomain helicase DNA binding protein 7) (71). BRG1 is essential for melanoma cell proliferation *in vitro* and for normal melanocyte development *in vivo* and combinations of MITF, SOX10, TFAP2A, and YY1 bind between two BRG1-occupied nucleosomes thus defining both a signature of transcription factors essential for the melanocyte lineage and a specific chromatin organization of the regulatory elements they occupy (71). Finally, recently published data of genome wide association study identified about 20 melanoma susceptibility loci, one of which was *MX2* – the HIV-1 restriction gene (72). It was shown that the level of *MX2* expression is critically mediated by YY1 further underlying the role of YY1 in melanoma (72).

However, still many questions arise regarding the exact role of YY1 in melanoma pathogenesis, progression, and drug resistance. A deeper characterization of YY1 molecular pathways of action may help to define novel biomarkers useful in melanoma prognosis. Future studies must investigate those molecular pathways to find more putative targets to develop innovative therapeutic approaches.

## Conclusions

In summary, YY1 seems to play a crucial role in many of the metabolic processes. Therefore, focusing on the detailed biology and administration of therapies that directly target YY1 or crosstalk pathways in malignant melanoma could facilitate the development of new and more effective treatment strategies and improve patients’ outcomes.

## Author Contributions

DK and AR contributed to the conception and design of the study. DK and EM performed the literature search. DK and EM wrote the first draft of the manuscript. AR finalized the manuscript. All authors contributed to manuscript revision, read, and approved the submitted version.

## Conflict of Interest

The authors declare that the research was conducted in the absence of any commercial or financial relationships that could be construed as a potential conflict of interest.

## Publisher’s Note

All claims expressed in this article are solely those of the authors and do not necessarily represent those of their affiliated organizations, or those of the publisher, the editors and the reviewers. Any product that may be evaluated in this article, or claim that may be made by its manufacturer, is not guaranteed or endorsed by the publisher.

## References

[1] Siegel RL Miller KD Fuchs HE Jemal A. Cancer Statistics, 2021. CA Cancer J Clin (2021) 71:7–33. doi: 10.3322/caac.21654 33433946

[2] Bandarchi B Jabbari CA Vedadi A Navab R. Molecular Biology of Normal Melanocytes and Melanoma Cells. J Clin Pathol (2013) 66:644–8. doi: 10.1136/jclinpath-2013-201471 23526597

[3] Garbe C Amaral T Peris K Hauschild A Arenberger P Bastholt L. European Consensus-Based Interdisciplinary Guideline for Melanoma. Part 1: Diagnostics - Update 2019. Eur J Cancer (2020) 126:141–58. doi: 10.1016/j.ejca.2019.11.014 31928887

[4] Passarelli A Mannavola F Stucci LS Tucci M Silvestris F. Immune System and Melanoma Biology: A Balance Between Immunosurveillance and Immune Escape. Oncotarget (2017) 8:106132–42. doi: 10.18632/oncotarget.22190 PMC5739707 29285320

[5] Shi Y Seto E Chang LS Shenk T. Transcriptional Repression Byyy1, a Human GLI Kruppel-Related Protein, and Relief of Repression by Adenovirus E1A Protein. Cell (1991) 67:377–88. doi: 10.1016/0092-8674(91)90189-6 1655281

[6] Austen M Luscher B Luscher-Firzlaff JM. Characterization of the Transcriptional Regulator YY1. The Bipartite Transactivation Domain Is Independent of Interaction With the TATA Box Binding Protein, Transcription Factor IIB, TAFII55, or cAMP-Responsive Element-Binding Protein (CPB)-Binding Protein. J Biol Chem (1997) 272:1709–17. doi: 10.1074/jbc.272.3.1709 8999850

[7] Kim JD Yu S Kim J. YY1 Is Autoregulated Through Its Own DNA-Binding Sites. BMC Mol Biol (2009) 10:85. doi: 10.1186/1471-2199-10-85 19712462 PMC2743690

[8] Yao YL Yang WM Seto E. Regulation of Transcription Factor YY1 by Acetylation and Deacetylation. Mol Cell Biol (2001) 21:5979–91. doi: 10.1128/MCB.21.17.5979-5991.2001 PMC87316 11486036

[9] Atchison M Basu A Zaprazna K Papasani M. Mechanisms of Yin Yang 1 in Oncogenesis: The Importance of Indirect Effects. Crit Rev Oncog (2011) 16:143–61. doi: 10.1615/CritRevOncog.v16.i3-4.20 PMC3417111 22248052

[10] Shi Y Lee JS Galvin KM. Everything You Have Ever Wanted to Know About Yin Yang 1. Biochim Biophys Acta (1997) 1332:F49–66. doi: 10.1016/S0304-419X(96)00044-3 9141463

[11] Gordon S Akopyan G Garban H Bonavida B. Transcription Factor YY1: Structure, Function, and Therapeutic Implications in Cancer Biology. Oncogene (2006) 25:1125–42. doi: 10.1038/sj.onc.1209080 16314846

[12] Donohoe ME Zhang X McGinnis L Biggers J Li E Shi Y. Targeted Disruption of Mouse Yin Yang 1 Transcription Factor Results in Peri-Implantation Lethality. Mol Cell Biol (1999) 19:7237–44. doi: 10.1128/MCB.19.10.7237 PMC84716 10490658

[13] Cho AA Bonavida B. Targeting the Overexpressed YY1 in Cancer Inhibits EMT and Metastasis. Crit Rev Oncog (2017) 22:49–61. doi: 10.1615/CritRevOncog.2017020473 29604936 PMC5955615

[14] Meliala ITS Hosea R Kasim V Wu S. The Biological Implications of Yin Yang 1 in the Hallmarks of Cancer. Theranostics (2020) 1:4183–200. doi: 10.7150/thno.43481 PMC7086370 32226547

[15] Sarvagalla S Kolapalli SP Vallabhapurapu S. The Two Sides of YY1 in Cancer: A Friend and a Foe. Front Oncol (2019) 9:1230. doi: 10.3389/fonc.2019.01230 31824839 PMC6879672

[16] Wang W Li D Sui G. YY1 Is an Inducer of Cancer Metastasis. Crit Rev Oncog (2017) 22:1–11. doi: 10.1615/CritRevOncog.2017021314 29604932

[17] Kim JD Faulk C Kim J. Retroposition and Evolution of the DNA Binding Motifs of YY1, YY2 and REX1. Nucleic Acids Res (2007) 35:3442–52. doi: 10.1093/nar/gkm235 PMC1904287 17478514

[18] Wai DC Shihab M Low JK Mackay JP. The Zinc Fingers of YY1 Bind Single-Stranded RNA With Low Sequence Specificity. Nucleic Acids Res (2016) 44:9153–65. doi: 10.1093/nar/gkw590 PMC5100589 27369384

[19] Weintraub AS Li CH Zamudio AV Sigova AA Hannett NM Day DS. YY1 Is a Structural Regulator of Enhancer-Promoter Loops. Cell (2017) 171:1573–88.e1528. doi: 10.1016/j.cell.2017.11.008 29224777 PMC5785279

[20] Yang WM Inouye C Zeng Y Bearss D Seto E. Transcriptional Repression by YY1 Is Mediated by Interaction With a Mammalian Homolog of the Yeast Global Regulator RPD3. Proc Natl Acad Sci (1996) 93:12845–50. doi: 10.1073/pnas.93.23.12845 PMC24008 8917507

[21] Lee JS Galvin KM See RH Eckner R Livingston D Moran E. Relief of YY1 Transcriptional Repression by Adenovirus E1A Is Mediated by E1A-Associated Protein P300. Genes Dev (1995) 9:1188–98. doi: 10.1101/gad.9.10.1188 7758944

[22] Kim JD Hinz AK Bergmann A Huang JM Ovcharenko I Stubbs L. Identification of Clustered YY1 Binding Sites in Imprinting Control Regions. Genome Res (2006) 16:901–11. doi: 10.1101/gr.5091406 PMC1484457 16760423

[23] Kleiman E Jia H Loguercio S Su AI Feeney AJ. YY1 Plays an Essential Role at All Stages of B-Cell Differentiation. Proc Natl Acad Sci (2016) 113(27):E3911–20.x. doi: 10.1073/pnas.1606297113 PMC4941496 27335461

[24] Banerjee A Sindhava V Vuyyuru R Jha V Hodewadekar S Manser T. YY1 Is Required for Germinal Center B Cell Development. PloS One (2016) 11(5):e0155311. doi: 10.1371/journal.pone.0155311 27167731 PMC4863967

[25] Hwang SS Kim YU Lee S Jang SW Kim MK Koh BH. Transcription Factor YY1 Is Essential for Regulation of the Th2 Cytokine Locus and for Th2 Cell Differentiation. Proc Natl Acad Sci (2013) 110(1):276–81. doi: 10.1073/pnas.1214682110 PMC3538243 23248301

[26] Josefowicz SZ Lu LF Rudensky AY. Regulatory T Cells: Mechanisms of Differentiation and Function. Annu Rev Immunol (2012) 30:531–64. doi: 10.1146/annurev.immunol.25.022106.141623 PMC6066374 22224781

[27] Hwang SS Jang SW Kim MK Kim LK Kim BS Kim HS. YY1 Inhibits Differentiation and Function of Regulatory T Cells by Blocking Foxp3 Expression and Activity. Nat Commun (2016) 7:10789. doi: 10.1038/ncomms10789 26892542 PMC4762897

[28] Mahmoud F Shields B Makhoul I Avaritt N Wong HK Hutchins LF. Immune Surveillance in Melanoma: From Immune Attack to Melanoma Escape and Even Counterattack. Cancer Biol Ther (2017) 18:451–69. doi: 10.1080/15384047.2017.1323596 PMC5639850 28513269

[29] Seidel JA Otsuka A Kabashima K. Anti-PD-1 and Anti-CTLA-4 Therapies in Cancer: Mechanisms of Action, Efficacy, and Limitations. Front Oncol (2018) 8:86. doi: 10.3389/fonc.2018.00086 29644214 PMC5883082

[30] Kwiatkowska D Kluska P Reich A. Beyond PD-1 Immunotherapy in Malignant Melanoma. Dermatol Ther (Heidelb) (2019) 9:243–57. doi: 10.1007/s13555-019-0292-3 PMC6522569 30927248

[31] Balkhi MY Wittmann G Xiong F Junghans RP. YY1 Upregulates Checkpoint Receptors and Downregulates Type I Cytokines in Exhausted, Chronically Stimulated Human T Cells. Iscience (2018) 2:105–22. doi: 10.1016/j.isci.2018.03.009 PMC6136936 30428369

[32] Hays E Bonavida B. YY1 Regulates Cancer Cell Immune Resistance by Modulating PD-L1 Expression. Drug Resist Update (2019) 43:10–28. doi: 10.1016/j.drup.2019.04.001 31005030

[33] Bonavida B Baritaki S. Dual Role of NO Donors in the Reversal of Tumor Cell Resistance and EMT: Downregulation of the NF-κb/Snail/YY1/RKIP Circuitry. Nitric Oxide (2011) 24:1–7. doi: 10.1016/j.niox.2010.10.001 20933602

[34] Bonavida B Baritaki S. The Novel Role of Yin Yang 1 in the Regulation of Epithelial to Mesenchymal Transition in Cancer *via* the Dysregulated NF-κb/Snail/YY1/RKIP/PTEN Circuitry. Crit Rev Oncog (2011) 16:211–26. doi: 10.1615/CritRevOncog.v16.i3-4.50 22248055

[35] Xia L Tan S Zhou Y Lin J Wang H Oyang L. Role of the Nfκb-Signaling Pathway in Cancer. Onco Targets Ther (2018) 11:2063–73. doi: 10.2147/OTT.S161109 PMC5905465 29695914

[36] Villanueva J Vultur A Lee JT Somasundaram R Fukunaga-Kalabis M Cipolla AK. Acquired Resistance to BRAF Inhibitors Mediated by a RAF Kinase Switch in Melanoma can be Overcome by Cotargeting MEK and IGF-1r/PI3K. Cancer Cell (2010) 18:683–95. doi: 10.1016/j.ccr.2010.11.023 PMC3026446 21156289

[37] Huerta-Yepez S Vega M Garban H Bonavida B. Involvement of the TNF-Alpha Autocrine Paracrine Loop, *via* NF-KappaB and YY1, in the Regulation of Tumor Cell Resistance to Fas Induced Apoptosis. Clin Immunol (2006) 120:297–309. doi: 10.1016/j.clim.2006.03.015 16784892

[38] Thomas WD Hersey P. TNF-Related Apoptosis-Inducing Ligand (TRAIL) Induces Apoptosis in Fas Ligand-Resistant Melanoma Cells and Mediates CD4 T Cell Killing of Target Cells. J Immunol (1998) 161:2195–200. 9725211

[39] Kumar R Herbert PE Warrens AN. An Introduction to Death Receptors in Apoptosis. Int J Surg (2005) 3:268–77. doi: 10.1016/j.ijsu.2005.05.002 17462297

[40] Vega MI Jazirehi AR Huerta-Yepez S Bonavida B. Rituximab-Induced Inhibition of YY1 and Bcl-xL Expression in Ramos Non-Hodgkin’s Lymphoma Cell Line *via* Inhibition of NF-κb Activity: Role of YY1 and Bcl-xL in Fas Resistance and Chemoresistance, Respectively. J Immun (2005) 175:2174–83. doi: 10.4049/jimmunol.175.4.2174 16081784

[41] Huerta-Yepez S Vega M Escoto-Chavez SE Murdock B Sakai T Baritaki S. Nitric Oxide Sensitizes Tumor Cells to TRAIL-Induced Apoptosis *via* Inhibition of the DR5 Transcription Repressor Yin Yang 1. Nitric Oxide (2009) 20:39–52. doi: 10.1016/j.niox.2008.08.001 18778787

[42] Hosseinahli N Aghapour M Duijf PHG Baradaran B. Treating Cancer With microRNA Replacement Therapy: A Literature Review. J Cell Physiol (2018) 233(8):5574–88. doi: 10.1002/jcp.26514 29521426

[43] Hongo F Garban H Huerta-Yepez S Vega M Jazirehi AR Mizutani Y. Inhibition of the Transcription Factor Yin Yang 1 Activity by S-Nitrosation. Biochem Biophys Res Commun (2005) 336(2):692–701. doi: 10.1016/j.bbrc.2005.08.150 16143308

[44] Shackleton M. Normal Stem Cells and Cancer Stem Cells: Similar and Different. Semin Cancer Biol (2010) 20:85–92. doi: 10.1016/j.semcancer.2010.04.002 20435143

[45] Fang D Nguyen TK Leishear K Finko R Kulp AN Hotz S. A Tumorigenic Subpopulation With Stem Cell Properties in Melanomas. Cancer Res (2005) 65:9328–37. doi: 10.1158/0008-5472.CAN-05-1343 16230395

[46] Brinckerhoff CE. Cancer Stem Cells (CSCs) in Melanoma: There’s Smoke, But Is There Fire? J Cell Physiol (2017) 232:2674–8. doi: 10.1002/jcp.25796 PMC5482763 28078710

[47] Kaufhold S Garbán H Bonavida B. Yin Yang 1 Is Associated With Cancer Stem Cell Transcription Factors (SOX2, OCT4, BMI1) and Clinical Implication. J Exp Clin Cancer Res (2016) 35:84. doi: 10.1186/s13046-016-0359-2 27225481 PMC4881184

[48] Schaefer SM Segalada C Cheng PF Bonalli M Parfejevs V Levesque MP. Sox2 Is Dispensable for Primary Melanoma and Metastasis Formation. Oncogene (2017) 36:4516–24. doi: 10.1038/onc.2017.55 28368416

[49] Varum S Baggiolini A Zurkirchen L Atak ZK Cantù C Marzorati E. Yin Yang 1 Orchestrates a Metabolic Program Required for Both Neural Crest Development and Melanoma Formation. Cell Stem Cell (2019) 24(4):637–53. doi: 10.1016/j.stem.2019.03.011 30951662

[50] Martinez-Ruiz GU Morales-Sanchez A Pacheco-Hernandez AF. Roles Played by YY1 in Embryonic, Adult and Cancer Stem Cells. Stem Cell Rev Rep (2021) 17:1590–606. doi: 10.1007/s12015-021-10151-9 PMC8553684 33728560

[51] Kim JY Lee JY. Targeting Tumor Adaption to Chronic Hypoxia: Implications for Drug Resistance, and How It Can Be Overcome. Int J Mol Sci (2017) 18:9. doi: 10.3390/ijms18091854 PMC5618503 28841148

[52] Ferrara N. VEGF and the Quest for Tumour Angiogenesis Factors. Nat Rev Cancer (2002) 2:795–803. doi: 10.1038/nrc909 12360282

[53] deNigris F Rossiello R Schiano C Arra C Williams-Ignarro S Barbieri A. Deletion of Yin Yang 1 Protein in Osteosarcoma Cells on Cell Invasion and CXCR4/angiogenesis and Metastasis. Cancer Res (2008) 68:1797–808. doi: 10.1158/0008-5472.CAN-07-5582 18339860

[54] Fu CY Wang PC Tsai HJ. Competitive Binding Between Seryl-tRNA Synthetase/YY1 Complex and NFKB1 at the Distal Segment Results in Differential Regulation of Human Vegfa Promoter Activity During Angiogenesis. Nucleic Acids Res (2017) 45:2423–37. doi: 10.1093/nar/gkw1187 PMC5389716 27913726

[55] LaGory EL Giaccia AJ. The Ever-Expanding Role of HIF in Tumour and Stromal Biology. Nat Cell Biol (2016) 18:356–65. doi: 10.1038/ncb3330 PMC4898054 27027486

[56] Zhang S Kim JY Xu S Liu H Yin M Koroleva M. Endothelial-Specific YY1 Governs Sprouting Angiogenesis Through Directly Interacting With RBPJ. Proc Natl Acad Sci USA (2020) 117(9):4792–801. doi: 10.1073/pnas.1916198117 PMC7060702 32075915

[57] Liu H Qiu Y Pei X Chitteti R Steiner R Zhang S. Endothelial Specific YY1 Deletion Restricts Tumor Angiogenesis and Tumor Growth. Sci Rep (2020) 10:20493. doi: 10.1038/s41598-020-77568-z 33235311 PMC7686504

[58] Zhao G Li Q Wang A Jiao J. YY1 Regulates Melanoma Tumorigenesis Through a MIR 9∈~∈RYBP Axis. J Exp Clin Cancer Res (2015) 34:66. doi: 10.1186/s13046-015-0177-y 26104682 PMC4511530

[59] Liu S Kumar SM Lu H Liu A Yang R Pushparajan A. MicroRNA-9 Up-Regulates E-Cadherin Through Inhibition of NF-κb1-Snail1 Pathway in Melanoma. J Pathol (2012) 226:61–72. doi: 10.1002/path.2964 22131135 PMC3959162

[60] Palmer MB Majumder P Cooper JC Yoon H Wade PA Boss JM. Yin Yang 1 Regulates the Expression of Snail Through a Distal Enhancer. Mol Cancer Res (2009) 7:221–9. doi: 10.1158/1541-7786.MCR-08-0229 PMC2819842 19208738

[61] Bhutia SK Mukhopadhyay S Sinha N Das DN Panda PK Patra SK. Autophagy: Cancer's Friend or Foe? Adv Cancer Res (2013) 118:61–95. doi: 10.1016/B978-0-12-407173-5.00003-0 23768510 PMC4349374

[62] Chang H Zou Z. Targeting Autophagy to Overcome Drug Resistance: Further Developments. J Hematol Oncol (2020) 13:159. doi: 10.1186/s13045-020-01000-2 33239065 PMC7687716

[63] Du J Ren W Yao F Wang H Zhang K Luo M. YY1 Cooperates With TFEB to Regulate Autophagy and Lysosomal Biogenesis in Melanoma. Mol Carcinog (2019) 58:2149–60. doi: 10.1002/mc.23105 31448838

[64] Kwiatkowska D Reich A. YY1 Is a Potential Key Player in the Pathogenesis of Malignant Melanoma. In: Bonavida B, editor. YY1 in the Control of the Pathogenesis and Drug Resistance of Cancer. London: Academic Press, Elsevier (2021). p. 163–9.

[65] Meliala ITS Hosea R Kasim V Wu S The Biological Implications of Yin Yang 1 in the Hallmarks of Cancer. Theranostics (2020) 10:4183–200. doi: 10.7150/thno.43481 PMC7086370 32226547

[66] Takeda T Tsubaki M Kato N Genno S Ichimura E Enomoto A. Sorafenib Treatment of Metastatic Melanoma With C-Kit Aberration Reduces Tumor Growth and Promotes Survival. Oncol Lett (2021) 22:827. doi: 10.3892/ol.2021.13089 34691254 PMC8527568

[67] Tang F Li S Liu D Chen J Han C. Sorafenib Sensitizes Melanoma Cells to Vemurafenib Through Ferroptosis. Transl Cancer Res (2020) 9:1584–93. doi: 10.21037/tcr.2020.01.62 PMC8798636 35117506

[68] Bonavida B Kaufhold S. Prognostic Significance of YY1 Protein Expression and mRNA Levels by Bioinformatics Analysis in Human Cancers: A Therapeutic Target. Pharmacol Ther (2015) 150:149–68. doi: 10.1016/j.pharmthera.2015.01.011 25619146

[69] Bevelacqua V Bevelacqua Y Candido S. Nectin Like-5 Overexpression Correlates With the Malignant Phenotype in Cutaneous Melanoma. Oncotarget (2012) 3:882–92. doi: 10.18632/oncotarget.594 PMC3478464 22929570

[70] Seberg HE Van Otterloo E Cornell RA. Beyond MITF: Multiple Transcription Factors Directly Regulate the Cellular Phenotype in Melanocytes and Melanoma. Pigment Cell Melanoma Res (2017) 30:454–66. doi: 10.1111/pcmr.12611 PMC5939569 28649789

[71] Laurette P Strub T Koludrovic D Keime C Le Gras S Seberg H. Transcription Factor MITF and Remodeller BRG1 Define Chromatin Organisation at Regulatory Elements in Melanoma Cells. Elife (2015) 4:e06857. doi: 10.7554/eLife.06857.025 PMC4407272 25803486

[72] Choi J Zhang T Vu A Ablain J Makowski MM Colli LM. Massively Parallel Reporter Assays of Melanoma Risk Variants Identify MX2 as a Gene Promoting Melanoma. Nat Commun (2020) 11:2718. doi: 10.1038/s41467-020-16590-1 32483191 PMC7264232

